# Role of serotonin receptor signaling in cancer cells and anti-tumor immunity

**DOI:** 10.7150/thno.55986

**Published:** 2021-03-11

**Authors:** Surojit Karmakar, Girdhari Lal

**Affiliations:** National Centre for Cell Science (NCCS), Ganeshkhind, Pune, MH-411007, India.

**Keywords:** 5-hydroxytryptamine, neuroimmune communication, neurotransmitter, serotonin receptor, serotonergic system

## Abstract

Serotonin or 5-hydroxytryptamine (5-HT) is a neurotransmitter known to affect emotion, behavior, and cognition, and its effects are mostly studied in neurological diseases. The crosstalk between the immune cells and the nervous system through serotonin and its receptors (5-HTRs) in the tumor microenvironment and the secondary lymphoid organs are known to affect cancer pathogenesis. However, the molecular mechanism of - alteration in the phenotype and function of - innate and adaptive immune cells by serotonin is not well explored. In this review, we discuss how serotonin and serotonin receptors modulate the phenotype and function of various immune cells, and how the 5-HT-5-HTR axis modulates antitumor immunity. Understanding how 5-HT and immune signaling are involved in tumor immunity could help improve therapeutic strategies to control cancer progression and metastasis.

## Introduction

**Figure 1:**
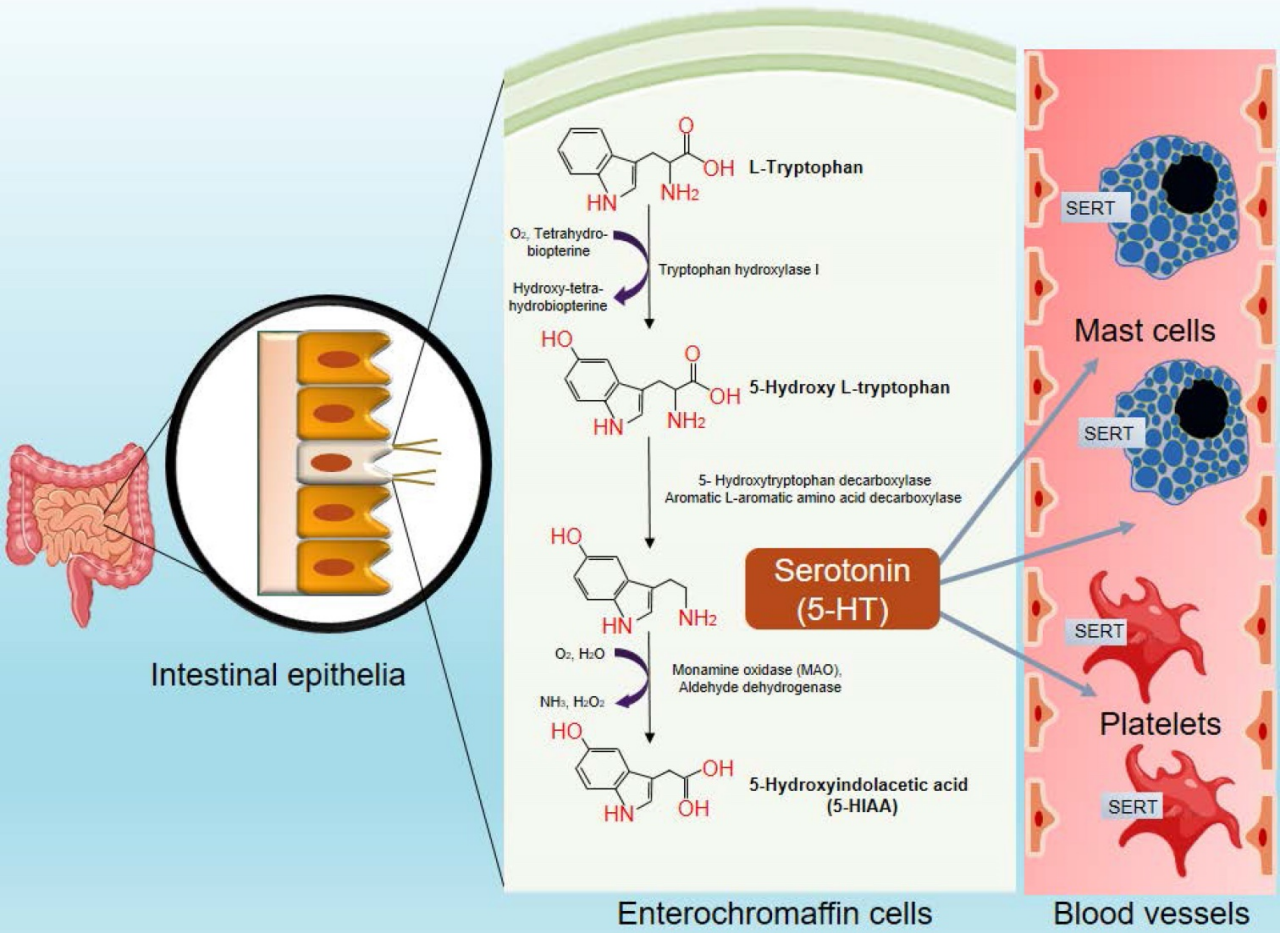
** Synthesis of peripheral serotonin.** Serotonin is synthesized from L-tryptophan by the TPH1 enzyme in the enterochromaffin cells of the intestinal epithelium, which is taken up by platelets and mast cells present in the local circulation. They release the serotonin elsewhere in the body either upon stimulation or due to their rupture.

Serotonin (5-Hydroxytryptamine; 5-HT) is a neurotransmitter that regulates signal transduction in the central nervous system (CNS). Apart from its neurotransmitter function, it also provides a link between neuronal signals and an immune response, and establishes a well-defined neuroimmune communication network in the body. Serotonin is synthesized form the essential amino acid L-tryptophan by a two-step process catalyzed by the enzymes, tryptophan hydroxylase (TPH) and monoamine decarboxylase (also known as 5-hydroxytryptophan decarboxylase or DOPA decarboxylase), as depicted in **Figure 1**. TPH is the rate-limiting enzyme of this serotonin biosynthetic pathway. TPH enzyme exists in two isoforms, TPH1 and TPH2. TPH1 is expressed widely in the periphery, with highest expression in the enterochromaffin cells of the intestine 1. The expression of TPH2 is restricted to neurons of the CNS. The existence of two isoforms of TPH indicates two different pools of serotonin in the body; one is restricted to the brain, and separated from the periphery by the blood-brain barrier, while the other pool is present in the peripheral systems. The enterochromaffin cells are the largest source of serotonin and accounts for almost 90-95% of the total serotonin production in the body. Mast cells and the brain are the second- and the third-largest sources of serotonin, respectively 2. The crucial role of serotonin pathway metabolites as a molecular mediator between gut and brain and its contribution in neuroimmune communications are reviewed recently 3, 4.

Serotonin synthesized in the brain performs various neuro-psychotic functions such as interventions via serotonergic neurons, regulation of appetite, sleep, mood, pain and sexual behavior 5. However, the serotonin produced in the periphery plays a wide array of functions such as vasoconstriction 6, regulating angiogenesis 7, controlling the bone density and osteoporosis 8, regulating diverse metabolic functions, including maintaining the blood glucose levels and obesity 9, and modulating gastrointestinal motility and gut microbiome content 10. Serotonin also functions as a growth factor, and modulates the cell cycle 11, inflammation and immunity 12. The serotonin synthesized by the enterochromaffin cells is secreted into the blood, most of which is taken up by the resident platelets and mast cells through the serotonin transporter (SERT) molecules present on their surface. The intracellular serotonin is then stored in dense granules at a high concentration of about 65 mmol/ml 12. Normal serotonin levels in the blood fall in the ranges of from 0.7 to 2.5 µM. Serotonin levels can rise to concentrations in the millimolar range in different pathological conditions, and at the synapses of serotonergic neurons following stimulation 13. It is released from the platelets and mast cells either upon their rupture or following crosslinking with IgE. After its physiological function is served, serotonin is degraded by the enzyme monoamine oxidase (MAO), resulting in the production of melatonin and 5-hydroxyindolacetic acid as metabolic byproducts 12.

**Figure 2:**
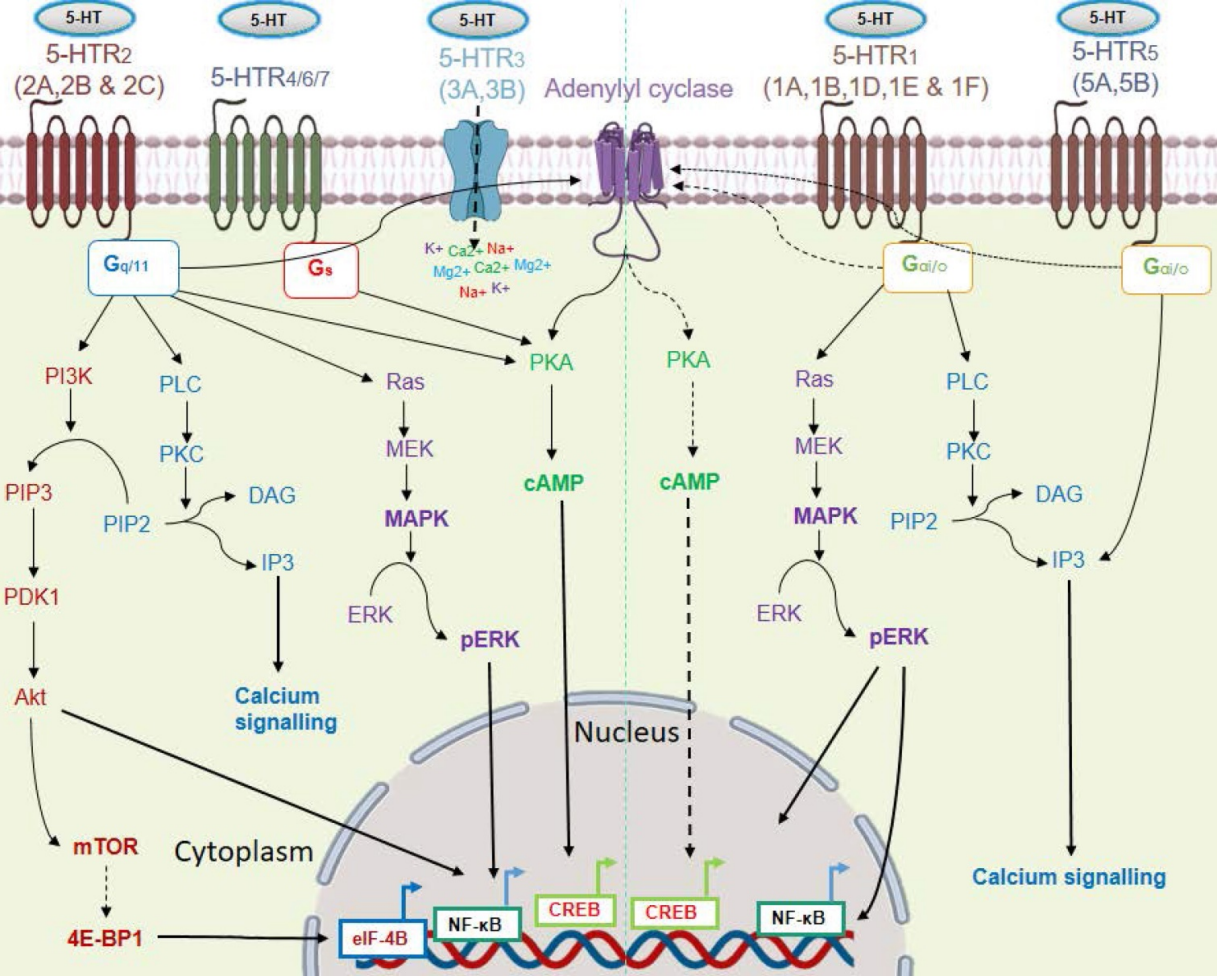
** Signaling pathways of the serotonin receptor (5-HTR) subtypes.** Serotonin signals through 15 receptor subtypes. Most of the receptors belong to GPCRs except the 5-HTR3 subfamily. These receptors activate four major interconnected signaling networks: PI3K/Akt, PKC/Ca^2+^, MAPK, and PKA-cAMP axis. Here, solid black arrows indicate stimulation of a pathway and black dashed arrows indicate inhibition of a pathway.

Response to serotonin is mediated via a wide repertoire of serotonin receptors and a serotonin transporter (SERT). At least 15 distinct subtypes of serotonin receptors (5-HTR_1-7_) are identified. Except for 5-HTR_3_, most of the 5-HTRs belong to the G protein-coupled receptor superfamily (GPCRs). The 5-HTR_3_ family of receptors (5-HTR_3A_ & 5-HTR_3B_) forms a ligand-gated non-specific cation channel, which permits the passage of cations like Na^+^, K^+^, Ca^2+^, and Mg^2+^ upon serotonin binding. Among all the 5-HTRs, the 5-HTR_1_ (5-HTR_1A-1F_) and 5-HTR_5_ (5-HTR_5A_ and 5-HTR_5B_) families of receptors are coupled with the intracellular G-protein Gα_i/o._ They inhibit adenylyl cyclase, resulting in subsequent downregulation of the PKA/cAMP activity 14. Signaling through 5-HTR_1A_ was also found to increase ERK phosphorylation in a PI3K/Akt-dependent pathway 15. The 5-HTR_2_ subtypes coupled with an intracellular stimulatory G protein (G_q/11_), which stimulates intracellular calcium signaling through the activation of phospholipase C. Serotonin also exerts its mitogenic activity through 5-HTR_2,_ via mitogen-activated pathway kinase (MAPK) signaling 16. 5-HTR_4/6/7_ triggers the PKA/cAMP axis via a stimulatory G protein (G_s_) 17. Signaling through its transporter involves entry of serotonin into the cells through SERT, followed by regulation of gene expression 18. Within the cell, transglutaminase 2 (TGM2) incorporates serotonin into the glutamine residue of histone H3, which subsequently helps in transcription of the target gene via recruitment of the general transcription factor II D (TFIID) 18. The various 5-HT/5-HTRs signaling pathways are shown in **Figure 2**. Due to its mitogenic property, serotonin also has a compelling role in cancer progression 19. In this review, we have discussed the role played by serotonin and the influence of serotonin-associated signaling on immune cells and cancer cells, and their implications in anti-tumor immunity.

## Effect of serotonin on various immune cells

**Figure 3:**
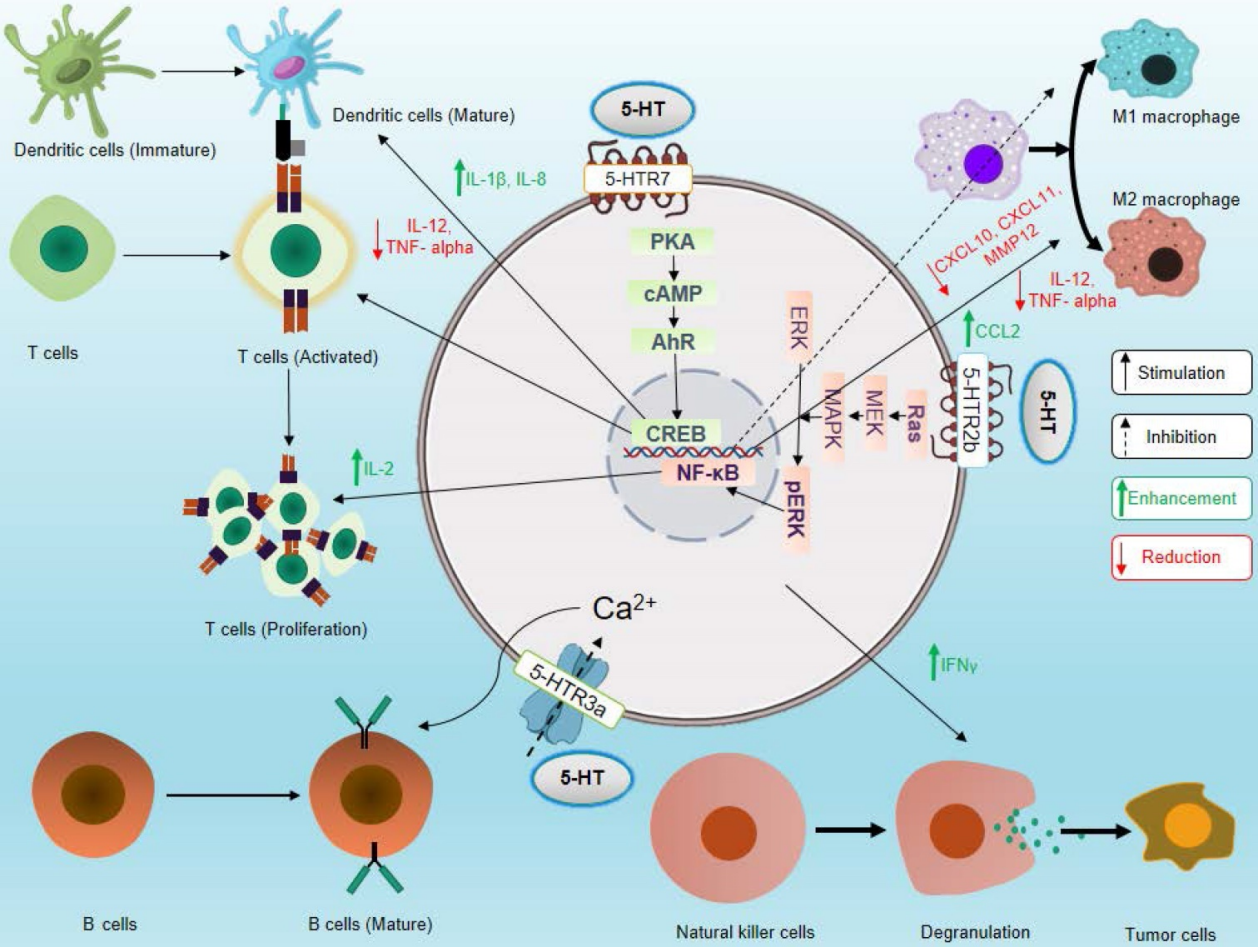
** Effects of serotonin signaling on different components of the immune system**. Serotonin signaling stimulates activation and proliferation of T cells, promotes maturation of DCs, supports B cell development, enhances cytotoxicity of NK cells, and stimulates polarization of macrophages toward the M2 phenotype. On the other hand, serotonin signaling inhibits M1 macrophage polarization. Here, solid black arrows indicate stimulation and black dashed arrows indicate inhibition, and green upward arrows indicate an increase in cytokine secretion, and red downward arrows indicate a decrease in cytokine secretion.

The role of serotonin in neuroimmune circuits in inter-organ communication and in inflammation and immunity is a new emerging area 20-22. Several types of immune cells express the serotonergic components and modulate the effector and regulatory functions of immune cells. Recently, it has been shown that IL-33 is sensed by the enterochromaffin cells, which induces the release of 5-HT, in turn activates the enteric neurons, thus serving as a link for neuroimmune communication 23. The effects of 5-HTRs signaling on various immune cells are depicted in **Figure 3**. The influence of serotonergic signaling on different immune cells and its importance in immunity are discussed below.

### Macrophages

Macrophages are professional antigen-presenting cells (APCs) that form a bridge between the innate and adaptive immune systems. These cells process antigens and present antigenic peptides through MHC molecules to activate T cells. During development, depending on the nature of the microenvironment, macrophages can develop into either the pro-inflammatory (M1) or the anti-inflammatory (M2) phenotype 24. While inducible nitric oxdide synthase (iNOS)^+^ M1 macrophages show pro-inflammatory properties 25, 26, M2 macrophages play a substantial role in the suppression of inflammation. M2 macrophages express arginase 1 (Arg1), which hydrolyses the amino acid L-arginine into urea and ornithine. The latter molecule is a precursor for L-proline and polyamines that play an important role in tissue repair, fibrosis and wound healing 27. Tumor-associated macrophages (TAMs) also secrete anti-inflammatory cytokines like TGF-β and IL-10, which help in promoting the development of regulatory CD4^+^FoxP3^+^ T cells, and prevention of an exaggerated immune response. TAMs also overexpress immune checkpoint proteins like PD-L1 and B7-H4, which suppress Cytotoxic T lymphocyte (CTL) activity 28.

Macrophages express 5-HTR_2B,_ 5-HTR_7_, and SERT 13, 29, 30. Serotonin receptor signaling through 5-HTR_2B_ in macrophages regulates the transcription of several genes (AP1, c/EBP, and SRF)_,_ which contributes to macrophage activation and polarization through phosphorylation of ERK1/2 molecules. 5-HTR_2B_ stimulation also promotes M2 macrophage polarization, but inhibits M1 macrophage polarization 30, 31. Casas-Engel *et al.* have demonstrated that 5-HTR_2B_ stimulation increased the expression of M2-specific markers (SERINB2, COL23AI, THBS1, and STAB1) and reduced the expression M1-specific markers (ALDH1, CD1B, and MMP12) 30. Serotonin also has an inhibitory effect on cytokine (TNF-α and IL-12) secretion by monocyte-derived M2 macrophages following lipopolysaccharide (LPS)-stimulation via the NF-κB pathway 30, 31. Serotonin and its metabolites (N-acetyl serotonin and melatonin) are known to reduce nitric oxide (NO) production in the human macrophage cell line RAW264.7 by virtue of their reactive oxygen species (ROS) scavenging activity 32. Serotonin impairs the expression of type-I IFN-dependent genes (CXCL10, CXCL11, IDO1, RSAP2, IL-27, and IFIT2) and stimulates TGF-β mRNA expression in the macrophages through the 5-HTR_7_-PKA axis 13. Serotonin also helps macrophages to acquire the profibrogenic and anti-inflammatory phenotypes through 5-HTR_7_, and lack of 5-HTR_7_ expression results in diminished macrophage infiltration and enhanced skin fibrosis pathology in mice 13.

### Dendritic cells (DC)

Though DCs are a relatively rare population of immune cells in the lymphoid organs, they are the most potent professional APCs that process and present antigens on their cell surface through MHC I and MHC II molecules, and activating CD8 and CD4 T cells, respectively 33. DCs are characterized into 3 different subtypes - (i) Conventional DCs (cDCs), (i) Plasmacytoid DCs (pDCs), and (iii) Monocyte-derived or inflammatory DCs (MoDCs or iDCs). Two main subtypes of the DCs, type 1 cDCs (CD103a^+^ cDCs) and type 2 cDC (CD11b^+^ cDC2) develop from a common DC precursor 34. pDCs are known to secrete type I IFNs and other tolerogenic factors such as TGF-β, IDO1 and IL-10, which promotes the induction of T cell anergy 34. Inflammatory DCs are developed from monocytes under inflammatory conditions and migrate to the site of inflammation. The role of these monocyte-derived DCs in cancer is not yet well characterized 33. Among the various subsets of DCs, type 1 cDCs are found to be systematically dysregulated in the early phase of pancreatic cancer, which leads to a poor prognosis 35. Loss of abundance of type 1 cDCs is also associated with poor response to antitumor immunity in pancreatic ductal adenocarcinoma (PDAC) 36. Type 1 cDCs are more potent activators of CD8 T cells and play a promising role in activating CTLs 33. Recently, CD103^+^ cDC1 were generated *in vitro,* and their use as an immunotherapeutic agent showed promising results in cancer 37.

DCs are known to express different subtypes of 5-HTRs in a maturity-dependent manner. Immature DCs express 5-HTR_1A_, 5-HTR_1E_, 5-HTR_2A_ and 5-HTR_3;_ whereas mature DCs express 5-HTR_2A_, 5-HTR_3,_ 5-HTR_4,_ and 5-HTR_7._ DCs are also known to express SERT and the serotonin-degrading enzyme, MAO-A 38, 39. Serotonin modulates cytokine secretion by DCs. It enhances the release of IL-1β and IL-8, and reduces the secretion of IL-12 and TNF-α from mature DCs by modulating a second messenger, cAMP, via 5-HTR_4_ and 5-HTR_7_ signaling. Serotonin also induces intracellular Ca^2+^ signaling in immature DCs via 5-HTR_1_ and 5-HTR_2_ signaling. Idzko *et al.* reported that IL-8 secretion is enhanced by serotonin through transcriptional regulation, whereas it modulates IL-1β secretion through a post-transcriptional regulation 38. Serotonin through 5-HTR_2B_ signaling, promotes the maturation of immature CD1a^+^ human monocyte-derived DCs upon TLR3 activation. Szabo *et al.* found that 5-HTR_2B_ signaling in moDCs upregulates the expression of DC maturation markers like CD80, CD83, and CD86 39. Serotonin also promotes the maturation and migratory property of bone marrow-derived DCs (BMDCs) via 5-HTR_7_-mediated activation of a small GTPase, Cdc45. CCL19-induced chemotaxis of BMDCs is also enhanced by serotonin, probably, via the upregulation of CCR7 expression on the surface of DCs 40.

The serotonin-mediated downregulation of IL-12 in DCs has a widespread effect on T cell polarization. It has been shown that serotonin reduces DC-mediated IFNγ^+^Th1 polarization and IL-17^+^Th17 polarization of CD4 T cells, whereas it enhances the IL-4^+^Th2 polarization 38. The expression of SERT on the surface of DCs enables them to take up peripheral serotonin and release it at immunological synapses formed between DCs and T cells, causing activation of T cells via 5-HTR_1_ signaling 41. Though serotonin stimulates the maturation of CD1a^+^ moDC and BMDC, it suppresses the secretion of pro-inflammatory cytokines by DCs. The effect of serotonin on cDCs and pDCs is not well defined and needs further investigation.

### Natural killer (NK) cells

NK cells are components of one of the most potent arms of the innate immune system against cancer. They impart its cytotoxicity against cancer cells by degranulation of cytolytic granules. Some cancer cells downregulate the expression of MHC I molecules on their surface, which helps them evade immune surveillance by CD8 cytotoxic T lymphocytes. However, NK cells can recognize the absence of MHC class I molecules on the surface of cancer cells and eliminate them 42, 43. Though NK cells mainly kill tumor cells through contact-dependent cytotoxicity, they can also contribute to anti-tumor immunity by secreting cytokines like IFN-γ and modulating the functions of the innate and adaptive immune cells and contribute to anti-tumor immunity 44. The effector functions of NK cells are characterized by the presence of activating receptors such as NKG2D, CD160, NKp46, NKp80, OX40, CD16 and 4-1BB, and inhibiting receptors such as NKG2A, KLRG1, CD161, TIGIT, CD96 and PD-1 on their surface 45. Inhibitory receptors such as NKG2A recognizes the presence of MHC class I molecules on the surface of target cells and prevents NK cell degranulation. Conversely, activating receptors like NKG2D interacts with ligands such as H60 (a-c), RAE (α-ε), and those from the MULT1 family in mice, and with ligand like MIC (MICA and MICB) and those from the ULBP1-6 family in humans, and promote NK cell activation and degranulation 42.

While there is no clear evidence of the expression pattern of the serotonergic system on NK cells 46, it was observed that the numbers of NK cells were substantially higher in patients with major depressive disorder, which increased further upon treatment with serotonin reuptake inhibitor (SSRI) antidepressants 47. Studies indicate that serotonin plays a role in enhancing the cytotoxicity, proliferation, and IFN-γ secretion potential of NK cells in the presence of autologous monocytes, through 5-HTR_2_ signaling 48, 49. This influence, which can be inhibited by IFN-α, is probably effected through its depolarizing activity on the plasma membrane of NK cells 50. Evans *et al.* showed that treatment with citalopram, an SSRI, enhanced NK cell-mediated cytotoxicity *ex vivo,* probably by causing an increase in the total available extracellular serotonin 51. In the presence of serotonin, NK cells can also lyse Daudi cells that are preferentially resistant to NK cell-mediated killing 52. In the tumor microenvironment, mononuclear phagocytes-like monocytes limit the cytotoxicity of NK cells by generating oxidative stress and releasing extracellular H_2_O_2_ and myeloperoxidase (MPO) 53. The free radical-scavenging ability of serotonin efficiently protects NK cells from the inhibitory effect of H_2_O_2_, and peroxidases like MPO 54, as well as MPO-independent serotonin-sensitive oxygen radicals 55. Though it has been shown that serotonin plays an important role in enhancing the cytotoxic potential of NK cells, the mechanism of serotonin signaling in these cells is not well studied, and needs to the explored.

### T cells

T cells form the major pillar of the adaptive immune system. Upon activation, CD4 T cells differentiate into Th1, Th2, Th9, Th17, or Treg cells 56, 57. CD8 T cells, on other hand, are the cytotoxic T lymphocytes (CTLs) that directly kill the target cells or antigen-expressing cells. Modern immunotherapeutic approaches have been designed to utilize the activities of effector T cells and suppress the regulatory T cells to eliminate cancer 58. In patients with major depressive disorder, the blood levels of pro-inflammatory cytokines (IL-2, IL-12, and TNF-α) and monocyte chemoattractant protein-1 (MCP-1) are relatively higher, while, the blood levels of anti-inflammatory cytokines like IL-4 and TGF-β are relatively lower 59. Reversal of this condition following treatment with antidepressants like SSRIs suggests that a role of serotonin plays a role in the modulation of cytokine secretion 59. Serotonin is known to impede TNF-α and IL-1β production in human peripheral blood mononuclear cells (PBMCs) through 5-HTR_2A_ signaling 60. These studies suggested a strong correlation between blood serotonin levels and the inflammatory state of the body, which was also reflected in the Th1/Th2 balance 61. Further, it was found that T cells have the ability to synthesize, store, degrade, and respond to serotonin and have a functional serotonergic system 41, 62. The expression of components of the serotonergic system, such as TPH1, MAO-A, and VMAT1, is relatively higher in CD8 CTLs as compared to CD4 helper T cells 63, 64. The expression of these enzymes increases further upon activation, and T cells actively respond to the autocrine-produced serotonin 63.

Serotonin causes activation of T cells via 5-HTR_3_-mediated upregulation of intracellular Na^+^ concentrations 65. 5-HTR_3_ signaling also facilitates the proliferation of T cells 65. 5-HTR_1_-mediated activation of cAMP signaling also activates T cells 41. Leon-Ponte *et al.* showed that blocking *in vivo* synthesis of peripheral serotonin by para-chlorophenylalanine (PCPA) reduces the activation and proliferation of CD4^+^CD25^+^ T cells in the periphery 66. Serotonin acts through 5-HTR_7_ signaling synergistically with TCR signaling and activates T cells. 5-HTR_7_ signaling activates NF-κB through ERK1/2 phosphorylation, which eventually drives T cells to secrete IL-2 and proliferate 66. Serotonin also suppresses the ability of T cells to elicit delayed-type hypersensitivity reaction through 5-HTR_2_ signaling 67. Many of the effects of signaling through serotonin receptors on T cell function were described using various chemical agonists and antagonists of 5-HTRs, which are discussed in **Table 1** and **Table 2**. Though the role of serotonin signaling has been explored in T cells, the detailed mechanism of how this signaling influences the differentiation and functions of various subsets of T cells is not clearly understood.

### B cells

The presence of B cells along with CD8 T cells in the tumor microenvironment (TME) has been reported, proved to be a better prognostic marker in many types of cancers, including melanoma, breast cancer and ovarian cancer 68, 69. The ability of B cells to serve as antigen-presenting cells (APCs) may promote better outcomes through B cell-T cell cooperation 70. One of the major functions of B cells is to produce antibodies upon activation. Antibodies against neoantigens that are sometimes detected in the serum, are considered as a good prognostic marker in some cancers 71. Such antibodies can promote opsonization and boost antibody-mediated phagocytosis by macrophages, or ADCC-mediated killing of cancer cells by NK cells 72.

Both normal and neoplastic B cells in germinal center malignancies seem to express 5-HTR_3A_ 73. B cells express SERT and have the capacity to reuptake serotonin from the periphery 74. Higher numbers of B cells have been reported in major depressive disorder (MDD) patients, following treatment with anti-depressant SSRIs 47. Rinaldi *et al.* have shown that serotonin plays a role in B cell development through 5-HTR_3A_ signaling in the germinal centers 73. Serotonin also enhances the mitogen-stimulated proliferation of B cells 73, 75. Serafin *et al.* have described the role of serotonin in the apoptosis of the Burkitt lymphoma cell line 76. This may be mediated through SERT, since SSRI treatment completely abolished this effect 76. B cells are the major source of humoral immunity in the body, and the impacts of serotonin on B cells needs to be explored further. Understanding the detailed molecular mechanisms will give greater insights into the interactions between serotonin signaling and B cells, and their importance in immunity.

## Role of serotonin in cancer cells

Cancers originating in different tissues express different serotonin receptors, which are listed in (**Table 3**). The role of serotonin in various cancers is discussed below.

## Hepatocellular carcinoma

Hepatocellular cancer (HCC) cells express different serotonin receptors, as seen from HCC cell lines and tumor tissue sections from HCC patients (**Table 3**). It has been observed that serum and platelet-driven serotonin levels are higher in HCC patients than in normal individuals 77. Serum serotonin levels have also been shown to be upregulated in the chemical-induced HCC mouse model 78, suggesting its involvement in the prognosis and progression of HCC 77. Through the 5-HTR_1B_ and 5-HTR_2B_ signaling pathways, serotonin promotes the proliferation of Huh7 and HepG2 human HCC cell lines under serum-deprived conditions by phosphorylation of ERK1/2 via the MAPK pathway 16, 79. TPH1 knockout mice show defective peripheral serotonin levels and develop resistance to CCL4-mediated liver tumor formation, suggesting that serotonin may play an essential role in liver cancer development 80. Serotonin is also found to affect autophagy in cancer cells. Autophagy or “self-eating” is a process that enables the cells to degrade or recycle damaged organelles and aged protein for maintenance of cellular homeostasis 81. Autophagy plays a dual role in cancer cells by regulating both cell survival and death. It helps to promote cancer by enhancing their ability to sustain their high metabolic demand by recycling damaged organelles and proteins. On the other hand, autophagy also performs a tumor suppressive role via maintaining cellular homeostasis by reducing cellular damage resulting from cellular stress 81. In pancreatic ductal adenocarcinoma (PDAC), autophagy leads to the degradation of MHC class I molecules, thus reducing antigen presentation by the cancer cells. These molecular changes result in resistance to immune checkpoint therapy, which can be reversed by combined treatment with an autophagy inhibitor or anti-CTLA4 antibodies 82. Autophagy is regulated by the LC3β and ATG8 proteins. LC3β promotes membrane fusion, elongation, and sealing of the autophagosome during autophagosome biogenesis 83. Another protein, p62 or sequestome-1, acts as a receptor that targets specific cargoes to autophagosomes by interacting with LC3β. When the process of autophagy is disrupted, p62 accumulates in the cytoplasm as ubiquitin-containing aggregates, which indicates inhibition of the autophagy in the cell 84. In serum-deprived conditions, cancer cells survive by enhancing autophagy, marked by an increase in the LC3β levels and a decrease in p62 accumulation in the cytoplasm. Serotonin shows a somewhat paradoxical role in the regulation of autophagy in hepatocellular carcinoma. Soll *et al.* showed that serotonin inhibits autophagy in HCC cells 80. On the contrary, Niture *et al.* showed that serotonin stimulates autophagy and induces apoptosis and steatosis (abnormal retention of lipids within the cell) in HepG2 and SK-Hep1 human HCC cell lines 80, 85. 5-HTR_1B_ and 5-HTR_2B_ signaling promotes autophagy by upregulating LC3β and autophagy-related effector proteins like Beclin1, ATG3, 4EBP1, and s65 85.

**Figure 4:**
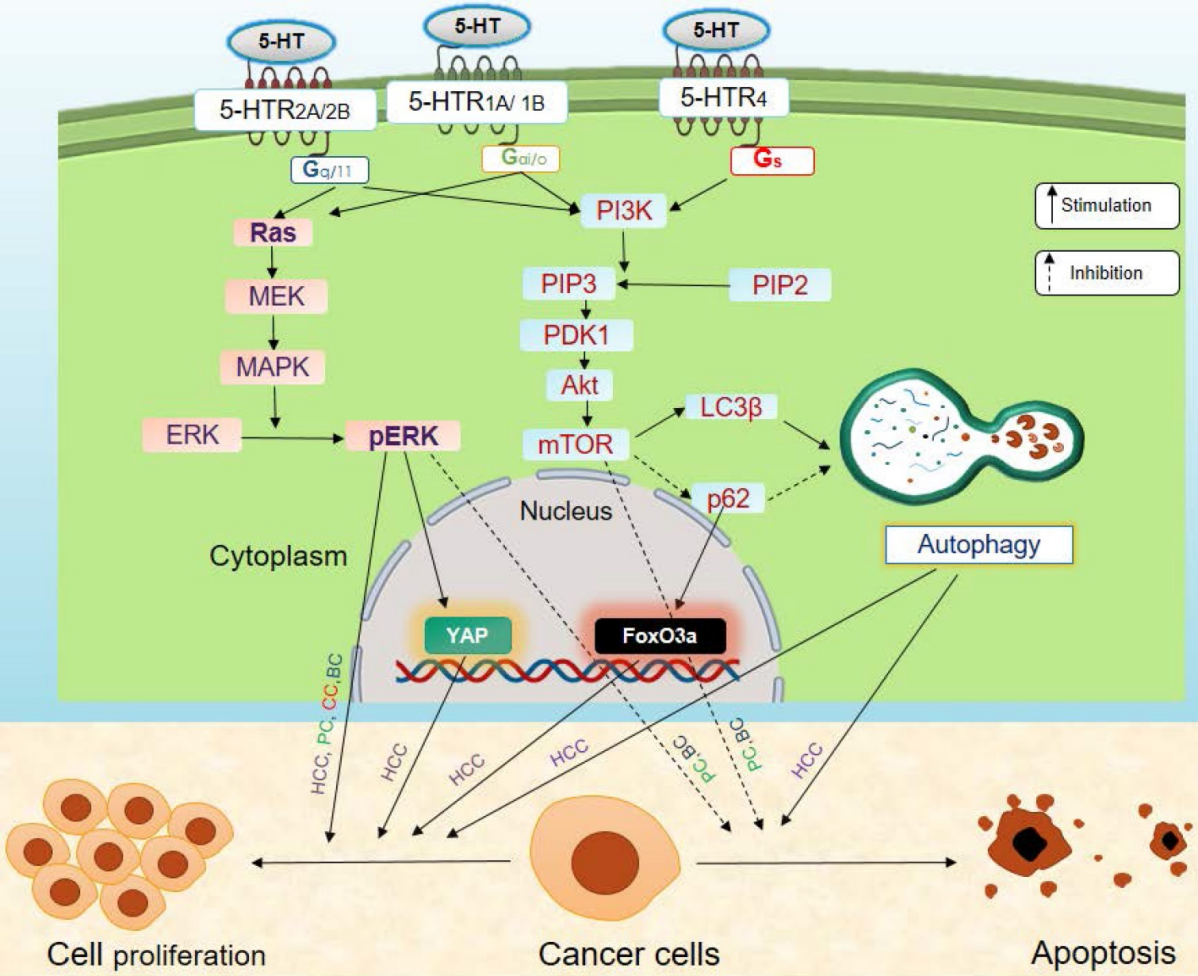
** Effects of serotonin signaling on cancer**. Serotonin signaling promotes tumor progression by stimulating proliferation and inhibiting apoptosis of cancer cells via MAPK and PI3K/Akt signaling axis. Here, solid black arrows indicate stimulation and dashed black arrows indicate inhibition. Abbreviations: BC, Breast cancer; CC, Colon cancer; HCC, Hepatocellular carcinoma; PC, Prostate cancer.

Serotonin modulates the development and progression of cancer via regulation of the oncogene Yes-associated protein (YAP), and the tumor suppressor, Vestigial-like family member 4 (VGLL4) protein 77, 86. Interestingly, YAP is an effector of the Hippo pathway that increases cell proliferation and decreases apoptosis in cancer cells 87. YAP binds to the transcription factor, TEF-1 and members of the abaA domain family member (TEAD). It promotes transcription of TEAD regulatory genes that are involved in increasing cell proliferation, suppressing apoptosis, and enhancing invasiveness and metastasis of cancer cells via epithelial-mesenchyme transition (EMT) (**Figure 4**). On the other hand, VGLL4 is a transcriptional cofactor that acts as a tumor suppressor. It binds to TEAD and inhibits the interactions between TEAD and YAP, thus suppressing TEAD-transcribed gene expression 88. An increase in the YAP/VGLL4 ratio is associated with cancer progression. Peripheral serotonin increases the YAP/VGLL4 ratio via phosphorylation of ERK through 5-HTR_2B_ signaling (**Figure 4**). This 5-HTR_2B_-pERK-YAP axis promotes the progression of tumors 77, 86. Serotonin also increases expression and phosphorylation of the oncogene FoxO3a in nutrition-starved Huh7 cells, through 5-HTR_2B_. FoxO3a promotes the growth and proliferation of serum-deprived human HCC cells. Serotonin also promotes cellular survival by inhibiting apoptosis of HCC cells by stimulating Akt phosphorylation 37, 79. Serotonin is known to induce oncogenic Notch signaling in HepG2 and Sk-Hep1 human HCC cell lines, likely through 5-HTR_1B_ and 5-HTR_2B_. In turn, this increases the survival capacity of HCC cells 85.

## Prostate cancer

Various serotonin receptor subtypes are expressed on androgen-sensitive (PC3, DU145) and androgen-insensitive (LCaNP, hPCP) prostate cancer cell lines. Serotonin receptor subtypes are expressed at both the primary site and the metastatic sites in humans 89-91. Serotonin stimulates the growth and proliferation of human prostate cancer cell lines (PC3, DU-145, LCaNP, hPCP, etc.) 89-92. The proliferative effect of serotonin is more prominent in androgen-insensitive prostate cancer cells than in androgen-sensitive prostate cancer cells. Serotonin mediates its pro-proliferative effect on prostate cancer cells through 5-HTR_1A_, 5-HTR_1B_, 5-HTR_2B,_ and 5-HTR_4_ signaling 89-92. Serotonin modulates the mitogenic MAPK/ERK and PI3K/Akt signaling pathways in prostate cancer, and stimulates phosphorylation of ERK1/2 through the MAPK pathway in the PC3 prostate cancer cell line (**Figure 4**). This effect of serotonin is likely mediated through 5-HTR_1A_ signaling, though the possibility of the involvement of other receptor subtypes cannot be ruled out. Serotonin also increases the phosphorylation of Akt, with concomitant activation of the PI3K/Akt pathway in the DU-145 prostate cancer cells (**Figure 4**). This modulation of the Akt pathway by serotonin also suppresses apoptosis in prostate cancer cell lines, possibly by signaling through 5-HTR_1B_ 37, 90, 93. Together, these studies suggest that serotonin influences prostate cancer progression. However, there is no conclusive evidence of the modulation of the PI3K/Akt pathway in PC3 and LNCaP prostate cancer cell lines, since they have a constitutive activation of PI3K/Akt pathway due to the deletion of the tumor suppression gene, PTEN 15, 94.

## Cholangiocarcinoma (Bile duct carcinoma)

Cholangiocarcinoma is one of the most devastating intrahepatic and extrahepatic bile duct cancer 95. In cholangiocarcinoma, serotonin metabolism is found to be dysregulated. Higher levels of serotonin have been detected in the serum of cholangiocarcinoma patients, as compared to healthy individuals. This phenomenon can be explained by the fact that cholangiocarcinoma cell lines (Mz-Cha-1, HuH-28, HuCC-T1, CCLP-1, SG231, and TFK1) show higher expression of the serotonin biosynthetic enzyme (TPH1), and lower expression of the serotonin degrading enzyme (MAO-A) 96. Serotonin promotes the growth and proliferation of cholangiocarcinoma cells via 5-HTR_1A_, 5-HTR_2A_, 5-HTR_2B_, 5-HTR_4,_ and 5-HTR_6_ signaling 96. As cholangiocarcinoma cells can synthesize serotonin, they can also respond to serotonin in an autocrine manner. Alipini *et al.* demonstrated that blocking serotonin synthesis with the TPH1 blocker, PCPA, reduces the proliferation of cholangiocarcinoma cells 96. Blocking of TPH1 with PCPA also led to reduced tumor growth, increased area of tumor necrosis, and increased fibrosis in the tumor 96. McMillin *et al.* showed that an increase in the free serotonin levels in the circulation caused by treatment with antidepressant SSRIs (sertraline and fluoxetine) enhances tumor progression 96. These effects may be mediated through the interaction of SSRIs with the components of the TME, rather than with the cancer cells. Alpini *et al.* showed that serotonin treatment increase BrdU incorporation and cell cycle progression in Mz-ChA-1 cells, which can be blocked by the TPH1 inhibitor, PCPA 96.

## Colon cancer

In colon cancer, serotonin modulates cancer cell proliferation and angiogenesis, which ultimately culminates in tumor progression. Serum serotonin levels in colon cancer patients are elevated, suggesting that serotonin may be a potential prognostic marker for colon cancer progression 97. The human colon cancer cell line HT29 also shows the expression of 5-HTR_1_, 5-HTR_3_, and 5-HTR_4_ 98, 99. Serotonin exerts mitogenic and anti-apoptotic effects on the HT29 cells mainly through 5-HTR_1A_, 5-HTR_1B_, 5-HTR_3_, and 5-HTR_4_ 98-100. This effect is also mimicked in mice colon cancer cell lines (CT26 and MC38) 101. Nocito *et al.* showed that peripheral serotonin deficiency slows tumor growth in mouse models with colon cancer allografts, and that external serotonin administration can reverse this condition 101. However, this effect of serotonin is not a consequence of its direct influence on cancer cells. Instead, serotonin increases angiogenesis by decreasing matrix metalloproteinase 12 (MMP12) expression in tumor-infiltrating macrophages. MMP12 keeps angiogenesis in check by increasing the circulatory levels of angiostatin, a potent inhibitor of angiogenesis 101. In colon cancer, serotonin modulates cancer cell proliferation and angiogenesis that ultimately culminates in tumor progression.

Kannen *et al.* showed that treatment with the antidepressant fluoxetine (an SSRI) treatment inhibits the proliferation of the mouse colon cancer cell line HT29 102, and inhibits carcinogen, dimethyl hydrazine-induced colon cancer in mice. This effect of the SSRI is mainly mediated through its inhibitory effect on micro-vessel formation and reduction of angiogenesis in the TME 103, and by affecting the energy-generating machinery of the cancer cells 104. Further studies are required to explain the detailed mechanism of serotonin's effect on colon cancer.

## Lung cancer

Clinicians have observed significantly higher serum serotonin levels in lung adenocarcinoma patients with depression, as compared to individuals without depression. Additionally, the expression of serotonin receptor subtypes like 5-HTR_1A_ and 5-HTR_1B_ in tumor tissues are also higher in patients with depression 105. Serotonin also plays a significant role in tumor progression in mice. In the Lewis lung cancer (LLC) allograft model, mice deprived of peripheral serotonin show significantly reduced tumor growth. This suggests that serotonin has a role in lung cancer progression 101. Serotonin controls tumor growth by stimulating angiogenesis via modulation of the MMP12/angiostatin axis 101. Lung adenocarcinoma is also known to express 5-HTR_3A,_ and knockdown of this receptor reduces the proliferation of lung adenocarcinoma cells 106. Liu *et al.* showed a negative correlation between 5-HTR_1A_ and 5-HTR_1B_ expression in lung adenocarcinoma tumors in patients with depression, and the number of tumor-specific T cells as well as the ratio of CD8/CD4 T cells present in the TME. Patients with higher expression of intratumoral 5-HTR_1A_ and 5-HTR_1B_, exhibit more CD4^+^CD25^+^FoxP3^+^ T_reg_ cells, and higher levels of expression of PD-L1 and pSTAT3 molecules in the TME 105. These studies indicate that sustained depression leads to the development of an immunosuppressive tumor microenvironment. This is associated with higher tumor-favoring cytokine release and transition of effector T cells to the regulatory phenotype 105. These reports thus indicate the involvement of serotonin in both tumor growth and immune modulation of the TME.

## Breast cancer

High levels of expression of TPH1 in the human breast cancer cell lines MCF7 and T47D indicate that they can synthesize large amounts of serotonin 107. MCF7 and human breast cancer tissues express the serotonin receptor subtype 5-HTR_2A_ and 5-HTR_3A_ 108, 109. Human cancer tissues from ductal carcinoma and invasive lobular cancer also express many of serotonin receptor subtypes 110, which are listed in **Table 3**. Serotonin enhances the rate of proliferation of MCF7 cells via 5-HTR_2A_ and 5-HTR_3A_ signaling 108, 111. Inhibiting these receptors with chemical antagonists was found to be reduce the proliferation and induced apoptosis of the MCF-7 cell lines 108. The triple-negative breast cancer (TNBC) cell lines (MDA-MB-231, HCC-1395, and Hs578T) shows higher expression of TPH1 and exhibit a stronger response to serotonin via 5-HTR_7_ in an autocrine manner, as compared to hormone-sensitive cell lines (MCF7, T47D). 5-HTR_7_ signaling activates the adenylyl cyclase, MAP kinase, and PI3K/Akt pathways 107. A dysregulated serotonin system enable proliferation, EMT, and survival in cancer cells via suppression of apoptosis 107. Treatment with SSRI and TPH1 inhibitors has been reported to suppress the growth of breast tumor-initiating cells (BTIC), and synergizes with chemotherapy, inhibiting breast cancer xenograft growth in mice 112. Recently, Gwynne *et al.* showed that the 5-HTR_4A_ antagonist, SB699551, targets human breast tumor-initiating cells through the canonical Gα_i/o_-coupled pathway and the PI3K/AKT/mTOR axis 113.

Sola-Penna *et al.* showed that serotonin promotes proliferation of the MCF-7 and MDA-MB-23 cell lines, and inhibits apoptosis in these cells by promoting glycolysis and oxidative phosphorylation. This action is performed through 5-HTR_2A/2C_ signaling-mediated ERK1/2 and Akt phosphorylation, and HIF1α expression. Serotonin also stimulates mitochondrial biogenesis in MCF-7 and MDA-MB-23 cells through activation of the cofactor PGC1α via the PKA-cAMP axis, through 5-HTR_2A/2C_ signaling 114. These phenomena may ultimately help mitigate a more invasive and aggressive forms of metastatic breast carcinoma.

## Pancreatic cancer

Pancreatic ductal adenocarcinoma (PDAC) tissues show a dysregulated serotonergic system. Microarrays of PDAC tissues from mice as well as in humans show higher expression of the serotonin-synthesizing enzyme TPH1, and lower expression of the serotonin-degrading enzyme MAO-A, as compared to control pancreas tissues 115. This indicates increased serotonin synthesis and reduced serotonin degradation in PDAC tumors. Among 13 different subtypes of 5-HTRs, 5-HTR_1B_, 5-HTR_1D,_ and 5-HTR_2B_ were found to be overexpressed in PDAC tissues as well as in pancreatic cancer cell lines, as compared to normal pancreas, whereas the expression of other subtypes of 5-HTRs was almost undetectable 115, 116. Overexpression of 5-HTR_2B_ in PDAC is associated with a poorer prognosis 115, 117.

Jiang et al observed that serotonin provides protection to the pancreatic cancer cell lines (BxPC-3, HPAC, PANC-1, and SW1990) by increasing cell viability and decreasing cellular apoptosis 115. Serotonin promotes the Warburg effect in pancreatic cancer cell lines by promoting glycolysis. Serotonin also increases the expression of HIF1α and c-MYC in pancreatic cancer cells. The protective effect of serotonin through 5-HTR_2B_ signaling on pancreatic cancer cell lines is mediated via the PI3K/Akt/mTOR axis. This cancer-promoting effect has also been observed in the pancreatic cancer xenograft model, where the antagonism of this receptor decreased cancer growth and metastasis 115. In addition to 5-HTR_2B_ signaling, 5-HTR_1B_ and 5-HTR_1D_ signaling also promote the proliferation and viability of pancreatic cancer cells (PaCa). 5-HTR_1B_ and 5-HTR_1D_ also promotes the invasion and metastasis, and inhibits clonogenicity of PaCa pancreatic cancer cells 116. Serotonin promotes EMT by upregulating TCF8/ZEB1 and snail proteins that promote EMT. 5-HTR_1B_ and 5-HTR_1D_ signaling promote metastasis by regulating the uPA/MMP2 axis 116. This combinatorial action of 5-HTR_1B_ and 5-HTR_1D_ signaling is mediated through Src-focal adhesion kinase (FAK) and the transglutaminase 2 - NF-κB signaling axis 116.

## Ovarian cancer

5-HTR_1A_, 5-HTR_1B_, 5-HTR_2B_, and 5-HTR_4_ are expressed in the normal ovary as well as in ovarian malignancies. Henriksen *et al.* found that these receptors are overexpressed in benign and non-invasive form of tumors, whereas their expression is of these receptors were downregulated in more invasive forms of ovarian tumors 118. In addition to tumor sections, different ovarian cancer cell lines (SKOV3, HEYA8, 2774, ES2, TOV112D, OV90, SW626, UWB1.298, and CaOV3) also show overexpression of 5-HTR_1A_, 5-HTR_1B_, 5-HTR1D, and 5-HTR_2A_. However, only the SW626, UWB1.298, and CaOV3 cell lines show overexpression of 5-HTR_2B,_ as compared to normal ovarian cells 119.

Christensen *et al* showed that treatment with both serotonin and 5-HTR_2A_ agonist increases the proliferation and survival of SKOV3, CP20, and ES2 ovarian cancer cell lines. They also showed that injection of serotonin and the SSRI, sertraline, increases the tumor weight and Ki67 expression in the SKOV3 tumor model in athymic nude mice 119. These finding collectively indicate that serotonin signaling promotes ovarian cancer development.

## Melanoma

The abnormal overexpression of the transcript encoding 5-HTR_2B_ in uveal melanoma is considered as a predictive marker for the formation of liver lesions in the metastatic form of uveal melanoma 120. Le-Bel *et al*. revealed that this deregulated expression of 5-HTR_2B_ in the metastatic uveal melanoma cell line is mainly due to the inability of the proteasome to degrade 5-HTR_2B_ in the cells 120.

The human skin melanoma cell line IPC-298 shows a constitutive expression of 5-HTR_2A,_ 5-HTR_2B,_ and 5-HTR_2C_. In contrast to other studies, Muller *et al.* showed that serotonin enhances radiation-induced inhibition of melanoma cell proliferation. This was likely due to the release of serotonin from mast cells following their exposure to ionizing radiation 121.

Liu* et al.* showed that the use of the 5-HTR_4_ agonists, tegaserod, significantly reduces melanoma cell growth and induces apoptosis in these cells. Tegaserod also reduces the tumor volume in the mouse melanoma model. However, tegaserod mediates this action through a serotonin-independent signaling mechanism, where it blunts the phosphorylation of S6 through inhibition of the PI3/Akt/mTOR pathway 122.

## Glioblastoma

Glioblastoma, a cancer of glial cells in the brain, and it is one of the deadliest forms of cancer. Depression and psychological distress are associated with worse outcomes with respect to glioblastoma progression. Otto-Meyer *et al.* showed that the treatment of glioblastoma patients with SSRI, which is often prescribed to treat psychological illness in these patients, does not significantly affect the overall survival rate of those patients 123.

Glioblastoma cell lines (U-373 MG, U-138MG, U-87 MG, DBTRG-05MG, T98G, H4, CCF-STTG1 and Hs 683) express the 5-HTR_7_ receptor subtype 124. Glioma tissues also express the 5-HTR_5A_ subtype, but its expression of 5-HTR_5A_ is lower in high-grade glioma than in low-grade glioma 125. Lieb *et al.* showed that serotonin promotes IL-6 secretion by the U-373 MG astrocytoma cell line through activation of the 5-HTR_7_ signaling mediated p38 MAPK and protein kinase Cє pathways. This mechanism may facilitate tumor progression by promoting a pro-inflammatory microenvironment 126. Lu *et al.* showed that agonizing 5-HTR_5A_ through valerenic acid inhibits the growth of glioblastoma cells both *in vitro* and *in vivo,* by promoting the elevation of intracellular ROS levels and activation of AMPK. Through this mechanism, valerenic acid also inhibits the EMT and subsequent metastasis in glioblastoma patients 125.

## Urinary bladder cancer

Different subtypes of serotonin receptors are found to be expressed in the human urinary bladder cancer cell line HT1376 and human bladder cancer tissues. Serotonin exerts a mitogenic effect and increases the proliferative potential of HT1376 cells via 5-HTR_1A,_ and more potently through 5-HTR_1B_ 127. Hence, this study suggests using 5-HTR_1B_ antagonists as a therapeutic alternative for use in urinary bladder cancer 127.

## Placental cancer or choriocarcinoma

The expression of 5-HTR_2A_ is apparent on the trophoblast cancer cell lines JEG3 and BeWo, which show an enhanced proliferation by altering the cell cycle, when treated with serotonin treatment 128. This action of serotonin is mediated via the MEK-ERK1/2 and JAK/STAT3 pathways, through 5-HTR_2A_ signaling 128.

## Gastric cancer

El-Salhy showed that serotonin, in combination with octreotide (a mimic of somatostatin) and galanin (a neuropeptide encoded by the *GAL* gene, and expressed in the brain, spinal cord and gut), reduces viability and increases apoptosis of in the human gastric cancer cell line (AGS). This combination was also result in reduced the tumor growth *in vivo* 129.

## Role of serotonin in tumor immunity

The various effects of serotonin signaling on immune cells has made it a target for studies *vis-à-vis* tumor immunity. For example, it has been reported that 5-HTR_2B_ and 5-HTR_7_ signaling promotes the development of anti-inflammatory M2 macrophages, and the latter promotes tumor development 30, 31. Nocito *et al.* found that serotonin can also downregulate the expression of matrix metalloproteinase 12 (MMP12) in macrophages, and promote tumor progression by increasing angiogenesis in the mouse model of colon cancer 101. MMP12 has an anti-angiogenic effect as it cleaves plasminogen to the angiogenesis suppressor, angiostatin. Furthermore, 5-HTR_2B_ is over-expressed on M2 TAMs adjacent to VE-cadherin-positive endothelial cells in the tumor. This indicates that serotonin may promote angiogenesis by modulating of blood vessel formation, assisted by adjacent macrophages through 5-HTR_2B_ signaling 30. Serotonin promotes maturation and chemotaxis of BMDCs 39, 40. Serotonin signaling also promotes anti-inflammatory DC development, which in turn has the potential to skew T cell polarization towards the regulatory phenotype 41. Regulatory T cells normally suppress cytotoxic T cell activity, and in turn, promote tumor development 58. Duerschmied *et al.* have shown that platelet serotonin also inhibits the infiltration of innate immune cells like neutrophils and monocytes in the site of inflammation, and leukocyte rolling on endothelial cells is faster in the absence of peripheral serotonin 130.

On the other hand, serotonin has been found to enhance the cytotoxic potential of NK cells 48, 49, and as well as serotonin signaling through 5-HTR_3A_ and 5-HTR_7_ has been reported to promote T cell activation 66. However, the detailed mechanism of action of serotonin on NK cells, and of T cells polarization after serotonin signaling mediated activation is still obscure.

Taken together, most of the ways in which serotonin signaling influences immune cells indicates that it facilitates tumor development via suppression of anti-tumor immunity. Therefore, the use of different antipsychotic drugs and chemicals that have the potential to modulate the serotonergic system, could have important implications in modulation of tumor immunity.

## Influence of application of agonists and antagonists of serotonin receptors on antitumor immunity

Serotonin has a total of 15 receptor subtypes and through which it is found to modulate a wide array of functions on both immune cells, stromal cells, and cancer cells. The importance of these receptors in tumor and immune modulation have been studied using different chemicals that either stimulate or inhibit signaling via the different 5-HTRs. These chemical agonists and antagonists of serotonin receptors are immensely important in explaining the role of serotonin in tumor immunity effected through the different receptor subtypes. The use of various chemical agonists and antagonists in immunity and cancer research, and their effect on various immune cells and cancer cells are listed in **Table 1** and** Table 2**, respectively. Some of these agonists and antagonists are broad-spectrum, which means that they act on more than one receptor subtypes. Among different agonists, the 5-HTR_1A/7_ agonist 8-OH-DPAT, the 5-HTR_3_ agonist 2-methylserotonin, and the 5-HTR_4_ agonists, 2-MHT and 5-CI, enhanced the release of IL-8 and IL-1β from DCs 38. Other agonists like the 5-HTR_1B_ agonist AnHcl, 5-HTR_1E/1F_ agonist BRL5443, and the 5-HTR2 agonist DOI also showed a stimulatory effect towards DCs 38. The 5-HTR_2B_ agonist BW-723C86, and the 5-HTR7 agonist AS19 enhanced the anti-inflammatory property of macrophages 13, 31. The 5-HTR3 agonist 2-methyserotonin as well as the 5-HTR7 agonist AS19 helped in T cell activation and further proliferation 65, 66.

Among different antagonists, SB-269970 and SB-258729 that block serotonin signaling through 5-HTR_7,_ have been widely studied. These antagonists decrease the differentiation of anti-inflammatory M2 macrophages 13, 30, modulates the cytokine release from DCs 38, 40, and inhibit ERK signaling in T cells 66. The broad-spectrum antagonists of serotonin receptors, methylsergide and ketanserin inhibited the activation of DCs 38 and abolished the ability of T cells to generate the delayed type of hypersensitivity 67. Among the other antagonists used, the 5-HTR_1A_ antagonist SB-216641 showed an effect on T cells similar to SB-269970 and SB-258729 66.

It is clear from these studies that chemical agonists and antagonists of serotonin receptors are promising modulators of immune cells, and thereby have a strong potential for application in therapeutics for diseases like cancer.

## Conclusion and future perspective

We have discussed here the complex interactions of serotonin with its receptors expressed on immune cells and cancer cells. Until recently, it was thought that serotonin is mainly confined to regulating body functions through concomitant neurons in the CNS and the peripheral nervous system. But it is now evident that other cells like immune cells and cancer cells also synthesize, release, and respond to serotonin. Though contradictory roles of serotonin in regulating the functions of different immune cells have been reported, most studies support that serotonin plays a role in enhancing their anti-inflammatory functions, as evident from the rise in anti-inflammatory cytokine secretion by some cells of the innate immune system. This suggests that serotonin may have a pro-tumorigenic effect by enhancing tumor immune evasion via the generation of an anti-inflammatory microenvironment. Gaining further insight into the cellular and molecular mechanisms of 5-HT/5-HTRs axis-mediated alterations in the tumor progression could have promising implications in clinical prognosis and therapeutics.

Serotonin promotes cancer progression by directly affecting cancer cells through various mechanisms like promotion of proliferation through cell cycle progression, autophagy, and suppression of apoptosis. This underscores the need to give due consideration to the role of serotonin in controlling tumor growth and in promoting antitumor immunity. This could pave the way to explore the potential of molecules that modulate the serotonergic system in improving or creating new therapeutic interventions for cancer.

## Figures and Tables

**Table 1:** Effects of serotonin receptor agonists in different immune cells

Agonists	Receptors	Functions					References
		DC	Macrophage	T cells	NK cells	B Cells	
S14506	5-HTR_1A_	--	--	Suppresses the rise of intracellular cAMP level in response to ATP	--	--	133, 134
8-OH-DPAT	5-HTR_1A/7_	Enhances dose dependant secretion of IL-8 and IL-1β and suppressed IL-12 and TNF-α release from mature DCs.	--	--	Increases NK Cell cytotoxicity.	Increases proliferation of B cells, characterised by [^3^H] thymidine uptake and S phase transition.	38, 48, 75
(+)ALK	5-HTR_1A_	--	--	--	a) Stimulates NK cell mediated killing of NK insensitive Daudi cells in the presence of IL-1, TNF-α and monocyte b) Increases IFN-γ secretion in the presence of IL-2.	--	49, 52
AnHCL	5-HTR_1B_	Stimulates intracellular Ca^2+^ spike in immature DCs.	--	--	--	--	38
BRL5443	5-HTR_1E/1F_	Stimulates intracellular Ca^2+^ spike in immature DCs.	--	--	--	--	38
DOI	5-HTR_2_	Stimulates intracellular Ca^2+^ spike in immature DCs.	--	Inhibits TNF-α production from PBMC.	--	--	38, 60
BW-723C86	5-HTR_2B_	--	a) Stimulates M2 macrophage polarization by signalling through the ERK pathway b) Inhibits LPS stimulated TNF-α production through the NFκB pathway and stimulates LPS stimulated CCL2 production from monocyte-derived macrophage thus imparts anti-inflammatory effect through AhR activation.	--	--	--	31, 30
2-Methyl- Serotonin	5-HTR_3_	Enhances dose dependant secretion of IL-8 and IL-1β and suppresses IL-12 and TNF-α release from mature DCs.	--	Activates T cells via increasing intracellular Na^+^ and thus helping the progression of T cell from S phase to G2/M phase.	--	--	38, 65
2MHT	5-HTR_4_	Enhances dose dependant secretion of IL-8 and IL-1β and suppresses IL-12 and TNF-α release from mature DCs.	--	--	--	--	38
5-CI	5-HT_1/4/7_	Enhances dose dependant secretion of IL-8 and IL-1β and suppresses IL-12 and TNF-α release from mature DCs.	--	--	--	--	38
AS19	5-HTR_7_	--	a) Enhances the expression of PDE2A and THBS1 through PKA-cAMP axis and thus promotes anti-inflammatory gene profile. b) Mimics the effect of 5-HT mediated downregulation of TNF-α and IL-12p40 secretion.	Stimulates T cell proliferation alike 5-HT.	--	--	13, 66

**Table 2:** Effects of serotonin receptor antagonists on immune cells

Antagonists	Receptors	Functions				
		DC	Macrophage	T cells	B cells	References
(+) WAY 100135	5-HTR_1A_	--	--	--	Inhibits the proliferative effect of serotonin on B cells.	75
Methylseride, ketanserin	5-HTR_2_	Abolishes the 5-HT-mediated intracellular Ca^2+^ spike in immature DCs.	--	a) Inhibits the capabilities of T cells to generate delayed type of hypersensitivity. b) Abolishes the 5-HT-mediated reduction in IL-1β secretion in PBMC.	--	38, 60, 67
SB-204741	5-HTR_2B_	--	a) Inhibits M2 macrophage specific gene (STAB1 & SERPINB2) expression. b) Reduces the expression of MMP12 and enhances the CD1B mRNA expression.	--	--	30
RS-39604	5-HTR_4_	Inhibits the LPS mediated IL-8 and IL-1β release through 5-HTR4 stimulation from immature DCs.	--	--	--	38
SB-269970, SB-258719	5-HTR_7_	a) Inhibits the LPS mediated IL-8 and IL-1β release through 5-HTR7 stimulation from immature DCs. b) Abolishes the increase in the length of processes of mature DCs by 5-HTR7 agonism.	a) Inhibits 5-HT mediated upregulation of TGFβ1 mRNA in monocyte-derived macrophages. b) Dampens the effect of 5-HT on limitation of expression of type I IFN responsive gene via PKA-cAMP axis. c) Reverses the 5-HT mediated inhibitory effect on LPS-stimulated TNF-α and IL-12p40 release from monocyte-derived macrophage. d) Dampens the 5-HT-mediated upregulation of M2 macrophage specific gene expression (SERPINB2, THBSI, and COL23AI).	Inhibits 5-HT mediated phosphorylation of the ERK1/2 and Iκβα in T cells.	--	13, 30, 38, 40, 66, 30

**Table 3:** Serotonergic system in different cancer cells

Cancer type	Models used	Serotonin receptor (5-HTR) subtypes expressed	References
Hepatocellular Carcinoma	Human hepatocellular carcinoma cell lines (Huh7 and HepG2), human HCC tissues, and mouse xenograft models.	1A, 1B, 2B, 7	16, 77, 79, 85
Prostate Cancer	Prostate cancer cell lines (PC3, DU-145, and LCaNP), and human prostate cancer tissue.	1A, 1B, 1D, 2A, 2B, 2C, 4	89, 91, 93
Breast Cancer	Hormone responsive cell lines (MCF7 and T47D), Triple-negative breast cancer cell lines (MDA-MB-231, HCC-1395, and Hs578T), and human breast cancer tissues.	1B, 2A, 2B, 3A, 4, 7	111, 131
Colon Cancer	Mice colon cancer cell lines (CT26 and MC38), human colon cancer cell line (HT29), and human colon cancer tissue.	1A, 1B, 3, 4	98-101
Pancreatic cancer	Human pancreatic cancer cell lines (AsPC-1, BxPC-3, Capan-2, CFPAC-1, HPAC, PANC-1 and SW1990PANC-1 and MIAPaCa-2) and human pancreatic cancer tissue microarrays.	1B, 1D, 2B	115, 116, 132
Ovary cancer	Human ovarian cancer cell lines (SKOV3, HEYA8, 2774, ES2, TOV112D, OV90, SW626, UWB1.298 and CaOV3) and human ovarian cancer tissues	1A, 1B, 2A, 2B, 4	118, 119
Melanoma	Uveal melanoma cell line, human skin melanoma cell line (IPC-298)	2A, 2B, 2C	120, 121
Glioblastoma	Glioblastoma cell lines ((U-373 MG, U-138MG, U-87 MG, DBTRG-05MG, T98G, H4, CCF-STTG1 and Hs 683) and Human glioblastoma tissues.	5A, 7	124, 125
Lung Cancer	*In vivo* mouse model of Lewis lung cancer and patient lung adenocarcinoma samples with depression	1B	101, 105
Bile duct cancer (Cholangiocarcinoma)	Cholangiocarcinoma cell lines (Mz-Cha-1, Huh-28, Hucc-T1, CCLP-1, SG231, TFK1)	1A, 2A, 2B, 4, 6	96
Urinary bladder cancer	Human bladder cancer cell line (HT1376)	1A, 1B, 1D, 2A, 2B, 2C	93, 127
Placenta cancer (Choriocarcinoma)	Human trophoblast cell lines (JEG3 and BeWo)	2A	128
